# The Use of GDS-15 in Detecting MDD: A Comparison Between Residents in a Thai Long-Term Care Home and Geriatric Outpatients

**DOI:** 10.4021/jocmr1239w

**Published:** 2013-02-25

**Authors:** Nahathai Wongpakaran, Tinakon Wongpakaran, Robert Van Reekum

**Affiliations:** a Department of Psychiatry, Faculty of Medicine, Chiang Mai University, Kingdom of Thailand; b Department of Psychiatry, University of Toronto, Toronto, Canada; c Institute of Medical Sciences, University of Toronto, Toronto, Canada

**Keywords:** Geriatric Depression Scale, Elderly, Long-term care home, Thai

## Abstract

**Background:**

To assess the psychometric properties of the Thai version of the 15-item Geriatric Depression Scale (TGDS-15) when screening for major depression (MDD) among geriatric outpatients (GOs) and long-term care (LTC) home residents in Thailand.

**Methods:**

This was a cross-sectional study of 156 geriatric outpatients and 81 LTC home residents. All 237 participants were given a Mini-Mental State Examination, a MDD diagnosis according to the Mini-International Neuropsychiatric Interview, and completed a TGDS-15 questionnaire. Sensitivity, specificity, overall accuracy, and positive and negative predictive values were calculated. A comparison between the two groups was carried out. Differential Item Functioning (DIF) using logistic regression and factor analytic study were also applied.

**Results:**

Overall, 38.4% of the participants were found to have MDD. The TGDS-15 was found to perform better when used with the GOs than with the LTC home residents, revealing a sensitivity of 0.92 and a specificity of 0.87 in the GOs (cut-off score of ≥ 5), but a sensitivity of 100% and a specificity of 49% with the LTC home group (cut-off score of ≥ 8), when comparing only cognitively intact subjects. The negative predictive value (NPV) was very good for both groups, but the positive predictive value (PPV) for the GO group was much better than for those in the LTC group (83.3% vs. 31.2%). Seven uniform DIF items were found - 2 by gender and 4 by age. Cronbach’s alpha was higher for the GO group than for the LTC home residents. Factor analysis supported a two-factor solution, using the ‘depressed mood’ and ‘positive mood’ factors, which accounted for 46.55% of the total variance.

**Conclusions:**

The TGDS-15 scale was effective at screening for MDD in elderly cognitively intact Thais, those in both GO and LTC settings, as the sensitivity and NPV were shown to be very good in both groups. However, in the LTC setting, the low specificity and PPV found leads to the need for a further assessment to be carried among the potentially depressed individuals, based on the GDS results. Taking the factor analytic study into account, a more suitable version of the GDS should be developed.

## Introduction

The Geriatric Depression Scale (GDS) has long been used to screen for Major Depressive Episodes (MDE, or ‘depression’) [1-7]. The original version of GDS is comprised of 30 items, asks about respondents’ feelings, behaviors and ideas in relation to depression over the previous week [8], and has been used with Thai elderly people for nearly two decades [9]. Due to the relatively long length of the original version, shorter versions have been developed, ranging from fifteen items to only one item [7, 10-12]. Among these, the 15-item GDS [7] is the most commonly used as a geriatric depression screening tool, and performs as well as the original, longer version [13, 14].

Even though evidence suggests that GDS-15 differs little from GDS-30 in terms of its ability to detect depression, it has different capabilities according to the gender, settings and gold-standard diagnoses used (ICD or DSM), as well as the type of depression (Major, Minor or dysthymia). Mitchell AJ et al [14] conducted a meta-analytic study and revealed that across fifteen studies using the GDS-15, a corrected sensitivity of 84.3% was found (95% CI = 79.7-88.4%) along with a specificity of 73.8% (95% CI = 68.0-79.2%). When used with respondents suffering from significant cognitive impairment, the sensitivity fell to 70.2% (n = 3; 95% CI = 47.7-88.5%) while the specificity rose slightly to 74.5% (95% CI = 61.2-85.7%). When used in an LTC home, the sensitivity and specificity scores were 86.6% and 72.3% respectively, while when used with outpatients, the sensitivity and specificity scores were 82.2% and 74.5%. For a sub-analysis restricted to outpatients with major depression only, there was no difference in sensitivity found when compared to the 30-item scale (79.5%), but a lower specificity was generated (63.1%).

In terms of factor structure of GDS-15, Kim et al [12] conducted a meta-analytic review and found among Asian populations, that there was a high Cronbach’s alpha, but found there to be a wide range of factors - ranging from 2 to 6. Furthermore, a study by Malagouti et al [15] among elderly Iranian subjects found there to be two factors, while between two and four factors were found in a Chinese study, three to four in Japan, and three to six in Korea. These meta-analytic studies also showed strong evidence of language differences in terms of the factor structure of the GDS.

The GDS-15 has never been tested for its psychometric properties on a sample of elderly Thais, so the primary aim of this study was to assess the effectiveness of the 15-item GDS when screening for major depression among elderly male and female Thais across two different settings - a geriatric outpatients’ clinic (OPD) and a long-term care (LTC) home. It was further hoped that the results of this research might give an indication of the potential impact of language and cultural factors on GDS psychometric performance.

## Materials and Methods

This study was conducted in Thailand and involved a cross-sectional design. The study population of 237 was split into two sample groups, with the first sample group including 156 participants of 60 years of age or over at living in an OPD (between January and December 2011), who were observed for major depressive disorder, and the second including LTC home residents who were examined in 2011 as part of their annual assessment. Each participant was evaluated by the same trained clinical research nurse using the Mini Mental State Examination (MMSE) which a score in the unimpaired range after correction for age and education, and the Mini-International Neuropsychiatric Interview (MINI) tools. The criteria used to exclude patients included the presence of cognitive impairment, or the presence of other serious medical conditions that would have proved an obstacle to proceeding with the research procedure, for example, the presence of cardiopulmonary disease, feelings of disorientation and drowsiness, and severe pain. This serious medical condition was assessed and decisions made by the patient’s attending physician, and signs of co-morbidity were looked for as part of any Axis I disorder and in accordance with DSM-IV by a research nurse using MINI. Each participant completed the Thai version of the Geriatric Depression Scale-15 (TGDS-15) as developed by the authors [16]. For the OPD sample, participants were recruited asking two screening questions related to depression (these being: Have you felt depressed? And: Have you lacked interest in the past two weeks?). For this project, those participants who had cognitive impairment - as identified by the Mini-Mental Status Examination (MMSE) were excluded. Over the enrollment period, 256 residents were eligible, 31 were excluded because their MMSE score was below the cut-off point, 35 were excluded because they showed signs of co-morbidity and instability (as indicated in the exclusion criteria), 31 were not allowed to participate in the study and 3 had incomplete data. Therefore, in total, only 156 were included in the analysis. For the LTC group, recruitment was conducted over a one month period during 2011, covering 91 residents in total. Of these, 10 were ineligible because they had an unstable medical condition; for example, delirium, hearing loss or inability to communicate, leaving 81 to be included in the study. The study was approved by the Ethics Committee of the Faculty of Medicine at Chiang Mai University, and all residents of the LTC home gave their informed consent.

### The MINI

The Mini-International Neuropsychiatric Interview (MINI) instrument was used here as the standard for diagnosing DSM IV major depression [16-18] - as developed by Sheehan [19], while the Thai version was validated by Kittiratanapaiboon et al [20] The Thai version has kappa ranges of between 0.27 and 0.87 to indicate the presence of depressive disorders. The research nurse administered the MINI survey across all participants and was not aware of the results of the GDS carried out by each patient. The two assessments were kept ‘blind’ and independent of each other.

### The Thai version of GDS-15

To create the Thai version of GDS-15, the author (NW) was granted permission by the developer (Yesavage JA) to translate it from English into Thai. The translation process followed a translation and cultural adaptation process which included a forward translation carried out by a geriatric psychiatrist (the first author), plus a backward translation into English carried out by a bilingual school professor who had no prior knowledge of the questionnaire. The two versions produced were assessed and compared item by item, until a consensus between the authors and the bilingual translator was reached. Only minor discrepancies were found in the items, and the final draft was then checked for grammatical errors and used on a sample of 30 people who were not participating in the study. The results were satisfactory, as the respondents understood the questions, and a Cronbach’s alpha of 0.75 was produced.

### Data analysis

The two groups were compared in terms of any variances in percentages, means and standard deviations, with an unpaired t-test or Mann-Whitney test adopted as appropriate for this. A comparison of the test performances from the different versions was carried out using receiver operating characteristics’ analyses, including sensitivities and specificities. Optimal cut-off scores were determined using Youden’s Index [21]. Point estimates and 95% confidence intervals (CIs) were computed to assess the test performance characteristics across different cut-offs and against the gold standard (MDD, as assessed by the MINI).

The factor structure was examined using exploratory factor analysis and SPSS for windows software (version 17) [22], with any missing data identified. Two of the respondents had a small amount of missing data, and so this was replaced with the means of the missing variables. A principal component analysis method (PCA) with oblimin rotation was used due to the fact that the items were correlated. Descriptive statistics confirmed that the sample measures were generally normally distributed, as determined by acceptable skewness and kurtosis scores of less than ± 3, that is, in the ranges 0.087 to 1.00 for skewness, and -1.01 to -2.01 for kurtosis. In addition, no outliers were found.

The Kaiser-Meyer-Olkin Measure of Sampling Adequacy scores were 0.84 for the OPD setting and 0.78 for the LTC home setting, while both groups gave Bartlett’s Test of Sphericity P-level scores of less than 0.001, indicating that the sample size was adequate for analysis purposes. In addition, parallel analysis, a method used to find a suitable factor by comparing real data with parallel random data, was used to help estimate the possible retaining factors [23]. The factors retained using these methods are those whose eigen values are greater than the eigen values from the random data [24]. Vista-Paran software was employed here for the parallel analysis [25].

Reliability refers to the internal consistency of a measure in a multiple-item construct, and here construct reliability was assessed using Cronbach’s alpha, with the recommended cut-off for the co-efficient alpha being 0.70 [26]. Pearson’s correlation was used to find the level of concurrent validity between GSC and the other measurements.

DIF was analyzed by using logistic regression to predict the item response across genders (male, female), ages (≤ 75 or > 75) and the ordinal regression for education levels (0 = no education, 1 = elementary, 2 = more than elementary). An odds ratio of ≥ 2 or ≤ 0.5 was considered DIF.

## Results

In total, 237 people were recruited into the study. The mean age of the entire group was 71.45 ± 7.43, though the mean age was higher among the LTC home group than the OPD group (P < 0.001). In terms of gender, most participants were female in both groups, though the ratio was significantly higher in the OPD group (P = .021). In terms of marital status, there was a significant difference in the number of participants ‘living together’ between the two settings (P < 0.0001).

After diagnosis with MINI, 38% were found to have major depressive disorder, with no difference between the two groups. The mean and SD scores for the MMSE were significantly higher in the OPD group than in the LTC group (23.96 ± 4.76 for OPD and 18.18 ± 6.98 for LTC, with P < 0.001). The mean and median MMSE scores across the total group were 23.46 ± 4.81 and 24.00 respectively. The mean GDS-15 score for the whole group was 6.41, with an SD of 3.82. The mean GDS-15 score was significantly higher for the LTC home group than it was for the OPD group. The clinical characteristics of the sample are shown in Table 1.

**Table 1:** Clinical Characteristics of the Sample

	Outpatients’ group (n = 156)	LTC group (n = 81)	All (n = 237)	Statistic	P-value
Age, Mean ± SD	68.83 ± 6.04	76.50 ± 7.28	71.45 ± 7.43	t = 8.61	< 0.0001
Gender, %female	106 (67.9)	45 (55.6)	151 (63.7)		0.021*
Marital Status: living with spouse, n (%)	79 (50.6)	6 (7.1)	35 (14.8)		< 0.0001*
Education (years, Mean ± SD)	3.30 ± 3.33	3.52 ± 3.56	3.46 ± 3.37	t = 0.465	0.643
MDD†, n (%)	68 (43.6)	23 (28.4)	91 (38.4)		0.088*
MDD: %Male:%female	12.2:34.1	10:19	11.4:27.0		
Cognitively impaired by MMSE, n (%)	0 (0)	28 (34.6)	-		
MMSE, Mean ± SD (Min-Max)	23.96 ± 4.76 (10 - 36)	18.18 ± 6.98 (3 - 36)	23.46 ± 4.81 (10 - 36)	t = 7.53	< 0.0001
TGDS-15, Mean ± SD	5.27 ± 3.78	8.59 ± 2.80	6.41 ± 3.82	t = 6.43	< 0.0001

*Fisher Exact; †diagnosis made by MINI; LTC: long-term care facility; SD: standard deviation; TGDS: Thai version of Geriatric Depression Scale; MMSE: Mini-Mental State Examination.

Table 2 shows that in the OPD sample, the TGDS-15 had a higher level of accuracy. When using a cut-off of ≥ 4, it yielded a sensitivity of 95% and a specificity of 75%, whereas when using a cut-off of ≥ 5, it yielded a sensitivity of 92% and a specificity of 87%. When using a cut-off of ≥ 6, it yielded a sensitivity of 86% and a specificity of 91%. Youden’s index indicated that a cut-off of ≥ 6 was optimal. For the LTC residents, with a cut-off of ≥ 8, the TGDS-15 yielded the highest sensitivity value of 100%, plus a specificity of 49%, whereas with a cut-off of ≥ 9 it gave a sensitivity of 80% and a specificity of 65%, and a cut-off of ≥ 10 gave a sensitivity of 60% and a specificity of 84%. Youden’s index slightly favored a cut-off score of ≥ 8. When all cases were accounted for, a cut-off of ≥ 7 yielded the best index of 0.63. The area under the curve was 0.82 (fair accuracy) for the LTC group, when compared to 0.96 for the OPD group and 0.88 for all participants. For all 209 cases, a cut-off score of ≥ 7 was given, which corresponded with the highest Youden index and yielded a positive predictive value or PPV (chance of having MDD when the screening is positive) of 66%, and a negative predictive value or NPV (the chance of not having MDD when the screening is negative) of 91.7%. Notably, the PPV for the OPD group was much higher than that for the LTC group (83.3% vs. 31.2%); whereas, the NPV values were similar across groups.

**Table 2:** GDS-15 Outcomes for the Outpatients’ Clinic and LTC Home Among Samples Without Cognitive Impairment

Setting	N	Alpha	AUC	95% Confidence Interval		Cut-off	Sensitivity	Specificity	Youden’s Index
				Lower Bound	Upper Bound				
Outpatients’ Clinic	156	0.84	0.96	0.93	0.98	≥ 4	95	75	0.70
						≥ 5*	92	87	0.80
						≥ 6	86	91	0.77
LTC home	53	0.7	0.82	0.69	0.94	≥ 8*	100	49	0.49
						≥ 9	80	65	0.45
						≥ 10	60	84	0.44
All cases	209	0.82	0.88	0.83	0.92	≥ 6	93	63	0.57
						≥ 7*	88	75	0.63
						≥ 8	79	81	0.59

LTC: long term care facility; AUC: area under curve; *The best cut-off score as suggested by Youden’s index.

**Figure 1:**
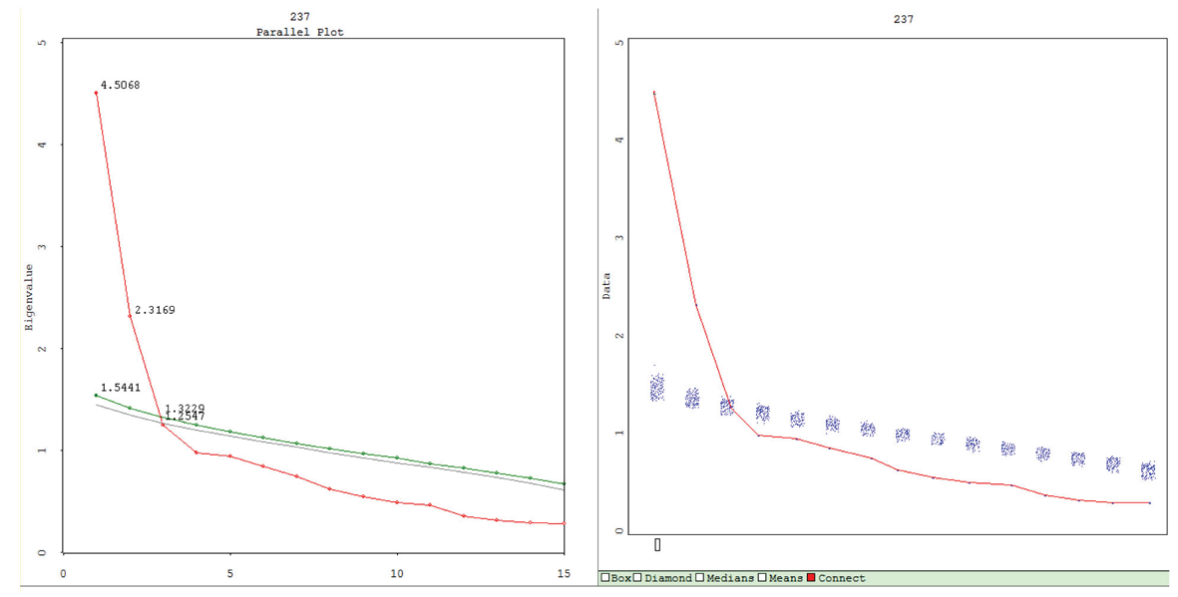
The plots of Scree Parallel (Left) and Scree Simulation (right) from parallel analysis. The Scree Parallel plot graphs the observed and estimated eigenvalues. Red indicates observed eigenvalues (4.5068 for Factor I and 2.3169 for Factor II), green indicates 95th percentile random data eigenvalues, and gray indicates mean of the random data eigenvalues. The lines intersect denotes the number of factors that must be retained according to the parallel analysis criterion. The lines cross at the second principal component. The Left graph is a summary of the Right, which provides greater detail on the simulation process. The Scree simulation plot demonstrates the Scree plot of the observed eigenvalues and all the Scree plots resulting from the simulated data (shown as a blue stripe). In this dataset, Factor I and Factor II are retained, factor III onwards have Eigen values less than that from simulations, and are therefore rejected.

In terms of internal consistency, it was found that Cronbach’s alpha for the entire questionnaire was good (Cronbach’s alpha = 0.82). Reliability analysis suggested only item 15 should be removed, as it yielded the lowest inter-item correlation (r^2^ = 0.126). With regard to factor structure (Table 3), four factors were extracted - with the first and second factors demonstrating acceptable Cronbach’s alpha scores of 0.84, 0.70 and 0.55 for Factors I, II and III respectively. There was a tendency towards a two-factor solution (Factors I and II) since the percentage of variance explained by Factors II and IV was low (less than 10%). This was reflected in the low Cronbach’s alpha (0.55) and zero Cronbach’s alpha scores calculated for Factor IV, since there was only one item. Parallel analysis also suggested a two-factor solution when using a Scree Parallel. In this dataset, Factors I and II were retained, while Factor III onwards had Eigen values less than those obtained from simulations, and were therefore rejected (Fig. 1). Table 3 shows the exploratory factor analysis scores from the TGDS-15 - indicating a two-factor solution. Item 15 was found to have the lowest loading on Factor I (0.245), with an unsatisfactorily poor communality value (0.062), whereas item 9 appeared to have double loadings on both factors (0.393 for Factor I and 0.295 for Factor II).

**Table 3:** Exploratory Factor Analysis Using TGDS-15: Two-Factor Solution (n = 209)

Item no.	TGDS item	h^2^	Factor I	Factor II
14	Feel that situation is hopeless	0.689	0.828	0.184
8	Feel helpless	0.675	0.821	0.164
12	Feel pretty worthless	0.693	0.817	0.284
4	Often get bored	0.556	0.745	0.134
3	Feel that life is empty	0.459	0.661	0.250
6	Afraid something bad is going to happen	0.417	0.621	
2	Have dropped many activities and interests	0.301	0.547	0.123
10	More problems with memory than most	0.195	0.432	0.160
15	Feel that most people are better off than me	0.045	0.184	
11	Think it is (not) wonderful to be alive	0.557	0.183	0.743
7	Feel (not) happy most of the time	0.62	0.387	0.738
5	(Not) in good spirits most of the time	0.557		0.718
1	Basically (not) satisfied with my life	0.512		0.699
13	Feel (not) full of energy	0.468	0.244	0.669
9	Prefer staying home to going out	0.24	0.304	0.428
	Eigenvalue		4.623	2.360
	Cumulated total explained, %		30.82	15.73
	Cronbach’s alpha, overall = 0.82		0.82	0.76

h^2^: communality loading coefficient less than 0.1 are not shown; TGDS: Thai version of Geriatric Depression Scale.

Of the 15 items evaluated, 6 showed evidence of bias, with odd’s ratios of ≥ 2.0 or conversely of ≤ 0.50 (Table 4). Uniform DIF was observed only for items 2 and 14 by gender, and for items 1, 4, 6 and 11 by age. No evidence of DIF was found in terms of the educational level.

**Table 4:** Differential Item Functioning: Odds Ratios for GDS-15 Items Across Categories for Gender, Education and Age

	Gender								Education								Age							
	uniform				non-uniform				uniform				non-uniform				uniform				non-uniform			

Item	OR	γ^2^	df	Sig.	OR	γ^2^	df	Sig.	OR	γ^2^	df	Sig.	OR	γ^2^	df	Sig.	OR	γ^2^	df	Sig.	OR	γ^2^	df	Sig.
1	1.528	35.229	2	0.214	0.969	35.343	3	0.738	0.916	34.003	2	0.658	1.015	34.073	3	0.791	2.21	37.435	2	0.018	1.082	38.036	3	0.446
2	0.418	62.254	2	0.018	1.039	62.382	3	0.719	1.266	57.449	2	0.259	1.033	57.716	3	0.606	0.562	55.941	2	0.103	0.83	59.07	3	0.071
3	0.391	93.18	2	0.699	0.955	93.28	3	0.754	1.118	92.68	2	0.644	1.035	92.837	3	0.692	1.187	90.082	2	0.661	1.023	90.103	3	0.885
4	1.059	93.611	2	0.886	1.102	94.195	3	0.44	1.332	95.316	2	0.227	0.933	96.035	3	0.401	0.4	96.09	2	0.023	0.885	96.811	3	0.384
5	0.848	29.668	2	0.613	0.932	30.29	3	0.436	1.109	29.555	2	0.591	0.931	31.244	3	0.198	1.874	30.769	2	0.055	1.065	31.191	3	0.521
6	1.558	51.76	2	0.216	0.934	52.195	3	0.516	1.573	54.651	2	0.036	0.998	54.653	3	0.97	0.336	59.46	2	0.004	0.986	59.474	3	0.907
7	1.086	103.007	2	0.84	1.066	103.231	3	0.633	1.026	102.619	2	0.915	0.93	103.351	3	0.397	1.511	100.172	2	0.296	0.991	100.176	3	0.949
8	3.198	127.321	2	0.035	0.967	127.338	3	0.898	0.985	122.761	2	0.957	1.006	122.762	3	0.966	1.014	120.649	2	0.976	0.808	121.529	3	0.335
9	0.773	54.537	2	0.476	1.002	54.538	3	0.983	0.788	55.609	2	0.256	0.975	55.769	3	0.69	0.71	54.268	2	0.344	0.997	54.268	3	0.982
10	1.479	49.858	2	0.257	0.793	54.54	3	0.045	1.013	48.782	2	0.948	1.008	48.8	3	0.891	0.925	48.468	2	0.82	0.945	48.468	3	0.585
11	0.646	64.51	2	0.24	0.904	65.295	3	0.386	0.805	64.01	2	0.321	1.023	64.12	3	0.741	2.097	64.796	2	0.04	1.333	69.117	3	0.061
12	1.104	157.542	2	0.864	1.471	158.994	3	0.213	0.812	158.306	2	0.528	0.761	159.949	3	0.202	1.183	155.716	2	0.745	0.743	156.511	3	0.362
13	0.801	66.64	2	0.542	0.912	67.308	3	0.424	0.987	66.529	2	0.952	1.059	67.289	3	0.385	1.558	64.896	2	0.215	0.825	68.037	3	0.071
14	5.169	66.64	2	0.01	1.242	67.308	3	0.483	0.66	140.373	2	0.194	0.793	141.911	3	0.216	0.581	137.781	2	0.282	1.83	139.795	3	0.23
15	0.967	9.336	2	0.922	0.99	9.349	3	0.907	0.982	9.412	2	0.929	1.016	9.495	3	0.773	0.998	8.495	2	0.995	1.146	10.276	3	0.196

Bold figures: Statistically significant, meets DIF criteria; OR: Odds Ratio; γ^2^: Wald Statistic; Df: Degrees of freedom; Sig: Significance level.

Based on this information, an analysis of the shortened GDS score was performed, deleting the 6 items that showed evidence of bias (items 1, 2, 4, 6, 11 and 14). The psychometric properties of this 9-item version (AUC = 0.849) were not a significant improvement over the original 15-item scale (AUC = 0.878; P = 0.48), in fact, the AUC was slightly lower than in the original version. Overall, DIF analyses suggested that age, level of education and gender did not have an effect on the measurement properties of the GDS-15 instrument using this sample.

## Discussion

Among the OPD group, the TGDS-15 gave the same results as it has in other studies, but this was not the case when used with the LTC home residents. When meta-analyzing the GDS-15, Mitchell et al [14] found no difference between outpatients’ clinics and LTC homes, while Rinaldi et al [27] and Blank et al [28] used the tool with multiple types of depression and yielded a better accuracy in an LTC home setting. Gerety et al [29] used the same criteria among major depression patients only, and yielded quite similar results to ours (a sensitivity of 88.2 and a specificity of 61.9). Moreover, the optimal cut-off score in their study was slightly higher (7/8) when compared to an average of 5/6 [13]. This contributed to the difference in characteristics seen between the two samples. One factor contributing to the low level of reliability in our study might have been the relatively high proportion of older people assessed, as well as the higher mean age of the LTC group when compared to the OPD group. We found, as in previous studies, that education level was not a source of bias when reporting depressive symptoms. It is worth noting that staying in an LTC home for the elderly in Thailand may be different in terms of social aspects than in other countries, due to the different cultural backgrounds. In general, elderly Thai people rarely move into an LTC home voluntarily, because it attracts stigma and means the residents are seen as having been abandoned by their offspring. These social values may affect how individuals view themselves as people, and how they respond to the GDS. One speculation the authors would make on this is that the depression found among those in an LTC home setting may be manifested more through physical than psychological symptoms [30, 31] - those captured by GDS, when compared with elderly people who live with their families. This may also explain why this group had a higher cut-off score here than the other group.

Kim et al [12] found that language differences may produce a different factor structure. They provided evidence for this when comparing mean variable cosines and congruence coefficients to assess the loadings of the factors. They found three common factors appeared consistently across most of the languages tested, these being: ‘dysphoria’ (items 3, 4, 8and 10), ‘social withdrawal-apathy-cognitive impairment’ (items 2, 12 and 14) - except in the Korean language, and ‘positive mood’ (items 1, 7, 9 and 15). The remaining factors were inconsistent across languages. Interestingly, a positive mood factor was found to apply to both the Anglo-Saxon and non-Anglo-Saxon respondents (namely, English; Japanese and Korean), though Kim et al stated that the reason why this happened was not clear.

Across the LTC home and OPD clinical Thai samples, our results yielded a two factor-solution for the GDS. The first factor comprised of items reflecting a ‘depressed mood’, while the second factor reflected a ‘positive mood’ (or negative items). This possibly supports what was found previously in a Turkish study with regard to positively and negatively worded factors [32]. Other items, such as ‘Feel that most people are better off than me’, ‘Prefer staying home to going out’ and ‘More problems with memory than most’, all of which have distorted loadings, not only failed to include an intended factor, but also obscured the true GDS factor structure.

In terms of item biases according to gender and education, we had similar results to Broekman, et al [33], who used different methods of study but found similar results for DIF with the following items: 1, 2, 4, 6, 11 and 14, but different types of bias. It would seem probable; therefore, that similar cultural background between the Chinese and Thai populations played a role. However, in terms of the factor structure, our study produced different results to theirs. Broekman et al [34] factor analyzed the GDS-15 using a large sample size and yielded a three-factor solution, with most items loaded on to Factor I (11 items) - with a Cronbach’s alpha of 0.83, two items loaded on to Factor II - with a Cronbach’s alpha of 0.32, and two items loaded on to Factor III - with a Cronbach’s alpha of 0.31. This suggested a one factor solution (loaded with 11 items), since the other two factors revealed a poor loading. In our study, 11 items within Factors I and II seem to have been reliable and so produced a clear two-factor structure. For Factor I, all 7 items represented the core symptoms of depression, whilst for Factor II this was due to the negatively worded items present, a phenomenon found in other measurement tools such as the Rosenberg Self-esteem Scale [35, 36]. Under Factor II, item 2 - ‘Have dropped many activities and interests’ and item 9 - ‘Prefer staying home to going out’, seem to have been less related to depression and more to the Thai way of life. Even though item 13 - ‘Feel (not) full of energy’, could be included in this category, it had a cross-loading with Factor II (negative wording). It is important to note that item 15 could not be merged with any other item, unlike in other studies such as Broekman et al [34]. Even though our study and Broekman et al’s shared the same Asian culture, the large difference in factor structure may be due to the fact that Broekman et al did not exclude respondents with cognitive impairment from their study.

In an attempt to shorten the GDS, Broekman et al removed the items with the lowest loading as well as those with DIF, and created GDS-7. However, two DIF items with high loading coefficients were retained (item 8 and 1). This shorter version showed excellent scaling and test performance. In our study, a 7-item version of the scale was created by excluding 5 negatively worded items, 2 items with a cross-loading and 1 item with a low inter-item correlation. The properties of this 7-item version showed improvements over the original GDS-15, with higher Cronbach’s alpha, AUC, PPV and NPV scores in both the OPD and LTC home settings (detailed data not shown here).

In summary, the Thai version of GDS-15 has been shown to work well as a major depression screening tool, and in accordance with the DSM-IV gold standard (as assessed by the MINI), in a geriatric outpatients’ clinic setting. The low specificity and PPV values produced by the GDS-15 in a Thai LTC home setting suggest the need for a further assessment to take place of the effectiveness of the Thai GDS-15 at diagnosing those potentially suffering from depression in Thailand. In terms of the factor structure, the GDS still needs to be revised and has not yet been stabilized across cultures (for example, Asian and Western). In addition, as a screening tool, a shortened GDS should be free of bias in relation to gender, culture and cognitive status, so further investigation needs to be carried out in this area.

### Limitations of the study and future research

Selection bias may have affected the results, plus the LTC home group was of a relatively small size. In addition, no test-retest was performed and this should be conducted in any future research studies. A shorter and more effective version of GDS; for example, one that is bias-free in terms of gender, age and education levels across various settings, particularly a culture-bias free version, should be developed and tested as part of any future studies.
